# The Additive Game: investigating methods to stabilize thin-layer cryo-EM specimens

**DOI:** 10.1063/4.0000832

**Published:** 2025-10-27

**Authors:** Mahitha Roy, Dominika Borek, Zbyszek Otwinowski

**Affiliations:** University of Texas Southwestern Medical Center, Dallas, TX, USA

## Abstract

In cryogenic electron microscopy single particle analysis (cryo-EM SPA), the sample-containing solution exists in an extremely high surface-to- volume ratio during the transitional period between post-blotting and plunge-freezing steps of sample preparation. The instability of ice presents an impediment to high-resolution structure determination because samples used for cryo-EM must be prepared as a thin-layer of sample- containing solution embedded in vitreous ice. The primary hazard a thin specimen is exposed to is diffusion- mediated adsorption to the air-water interfaces (AWIs). Adsorption has effects that undermine structure determination such as: 1) denaturation, 2) aggregation, and 3) preferred orientation. Severe preferred orientation will result in an anisotropic 3D reconstruction, which will result in a distorted map.

Our objective is to investigate the use of human apoferritin to stabilize thin-layer cryo-EM specimens. Apoferritin is a very stable molecule with a well-known structure that can be efficiently reconstructed. We hypothesize that the use of human apoferritin: (1) will stabilize the ice layer, (2) will not add additional noise, and (3) will provide contrast for motion correction. Adding apoferritin mixed with a target molecule may result in stabilization of the ice layer and modulation of preferred orientation.

Coproheme decarboxylase (ChdCs/HemQ) and glutamine synthetase (GS) were chosen as the target molecules because of their preferred orientation in cryo-EM experiments. We prepared grids of apoferritin mixed with ChdCs and GS in several molar ratios for cryo-EM SPA, and for cryogenic electron tomography (cryo-ET) using a 200 kV microscope. We analyzed the impact of apoferritin on the reconstruction of each target molecule, and the position of particles in the ice.

